# Ivosidenib for IDH1‐Mutant Intrahepatic Cholangiocarcinoma: Insights From a Multicenter Real‐World Study

**DOI:** 10.1111/liv.70295

**Published:** 2025-08-26

**Authors:** Monica Niger, Margherita Rimini, Florian Castet, Anna Melzer, Mario Domenico Rizzato, Tiziana Pressiani, Daniele Lavacchi, Giuseppe Aprile, Torsello Angela, Tiziana Saladino, Noventa Silvia, Pasqua Cito, Alessandro Pastorino, Eduardo Terán‐Brage, Carolina Sciortino, Silvia Camera, Chiara Pircher, Mara Persano, Vincenzo Mazzaferro, Silvia Foti, Kreina Sharela Vega, Thomas J. Ettrich, Lorenzo Antonuzzo, Lorenza Rimassa, Sara Lonardi, Lukas Perkhofer, Teresa Macarulla, Filippo Pietrantonio, Andrea Casadei‐Gardini

**Affiliations:** ^1^ Medical Oncology Department Fondazione IRCCS Instituto Nazionale dei Tumori di Milano Milan Italy; ^2^ Department of Oncology IRCCS San Raffaele Scientific Institute Hospital, Vita‐Salute San Raffaele University Milan Italy; ^3^ Gastrointestinal and Endocrine Tumor Unit Vall d'Hebron Institute of Oncology (VHIO), Hospital Universitari Vall d'Hebron Barcelona Spain; ^4^ Internal Medicine 1 University Hospital Ulm Ulm Germany; ^5^ Institute of Molecular Oncology and Stem Cell Biology Ulm University Hospital Ulm Germany; ^6^ Department of Oncology Veneto Institute of Oncology IOV‐IRCCS Padua Italy; ^7^ Medical Oncology and Hematology Unit Humanitas Cancer Center, IRCCS Humanitas Research Hospital Rozzano Italy; ^8^ Clinical Oncology Unit Careggi University Hospital Florence Italy; ^9^ Department of Oncology University and General Hospital Udine Italy; ^10^ UOC Oncology, AO San Giovanni Roma Italy; ^11^ Oncology Unit AST3 Macerata Hospital Macerata Italy; ^12^ Medical Oncology Fondazione Poliambulanza Istituto Ospedaliero Brescia Italy; ^13^ Oncologia Medica Ospedale San Pio Di Castellaneta Taranto Italy; ^14^ Medical Oncology Unit 1 IRCCS Ospedale Policlinico San Martino Genoa Italy; ^15^ Department of Oncology and Hemato‐Oncology University of Milan Milan Italy; ^16^ Fondazione IRCCS Istituto Nazionale Tumori HPB Surgery and Liver Transplantation Milan Italy; ^17^ Department of Biomedical Sciences Humanitas University Milan Italy

## Abstract

**Background & Aims:**

Cholangiocarcinoma (CCA) is a rare cancer with limited therapeutic options and a poor prognosis. While first‐line combination therapies have improved outcomes, second‐line treatment remains challenging. Ivosidenib, an *IDH1* inhibitor, has shown promise in treating *IDH1* mutant CCA, but real‐world data is limited. This study aims to evaluate ivosidenib's efficacy and safety in a large cohort of patients and compare it with second‐line chemotherapy.

**Methods:**

This observational, retrospective, multicenter study included patients with advanced *IDH1* mutant CCA treated with ivosidenib at 11 European institutions from May 2021 to September 2024. The primary endpoint was progression‐free survival (PFS); the main secondary objectives were overall survival (OS), disease control rate (DCR), overall response rate (ORR) and safety. As a pre‐planned exploratory objective, mPFS and OS of second‐line ivosidenib and FOLFOX/CAPOX were compared by means of inverse probability of treatment weights (IPTW)‐adjusted analysis.

**Results:**

The study included 46 patients treated with Ivosidenib; 43.5% received ivosidenib as second line and 56.5% as ≥ third line. Median PFS and OS were 3.7 (95% CI, 2.2–36.5) and 11.5 months (95% CI, 9.5–36.5). DCR was 50.0%. Grade ≥ 3 adverse events occurred in 8.7% of patients. IPTW‐adjusted mPFS was 6.9 months with ivosidenib and 2.1 months with FOLFOX/CAPOX (HR: 0.36, 95% CI, 0.20–0.64, *p* = 0.0005), while the mOS was 15.9 and 9.0 months with ivosidenib and FOLFOX/CAPOX, respectively (HR: 0.47, 95% CI, 0.23–0.96, *p* = 0.0405).

**Conclusion:**

This study suggests that ivosidenib is a valid option for patients affected by metastatic *IDH1* mutant CCA after at least one line of standard treatment.

**Summary**

- This study investigated the effectiveness and safety of ivosidenib, an *IDH1* inhibitor, in treating patients with a specific type of advanced bile duct cancer (cholangiocarcinoma) that has a mutation in the IDH1 gene.
- Data were collected from 46 patients across 11 hospitals in Europe.
- The study found that ivosidenib helped control the disease and extended survival, with manageable side effects.
- Additionally, when compared to standard chemotherapy, ivosidenib showed better outcomes.
- The results suggest that ivosidenib is a valuable treatment option for patients with *IDH1* mutant cholangiocarcinoma.

## 1 Introduction

Cholangiocarcinoma (CCA) is a rare cancer with limited therapeutic options and a poor prognosis [1, 2, 3]. Despite the positive results of the phase 3 trials TOPAZ‐1 and KEYNOTE‐966 with the first‐line combination of cisplatin, gemcitabine + PD‐(L)1 inhibitors (durvalumab or pembrolizumab, respectively) [4, 5], which are now considered the standard of care, patients with advanced disease still have an unsatisfactory median overall survival (mOS) of around 12 months [5, 6]. In terms of second‐line therapy, the standard option is chemotherapy with 5‐fluoruracil/leucovorin (5‐FU) and oxaliplatin (FOLFOX), after the ABC‐06 study showed its significant OS benefit over active symptom control [7]. However, given the poor results of this therapy, which yields 6.2 months of mOS and the mounting evidence for biomarker‐directed therapy for CCA, with several potentially actionable molecular targets reported in up to 47% of patients [8], international guidelines recommend performing next‐generation sequencing (NGS) for all patients potentially eligible for targeted therapy [9, 10, 11].

Among the most valuable targets, isocitrate dehydrogenase‐1 (*IDH1*) mutations can be found in ~15%–20% of CCA, mostly in intrahepatic CCA (iCCA) [12, 13] and lead to hyperproduction of the oncometabolite 2‐hydroxyglutarate (2HG) [14, 15], resulting in epigenetic dysregulation, aberrant cell metabolism and promoting tumourigenesis [16]. So far, the prevailing strategy to treat *IDH1* mutant cancers has been to suppress 2HG production with inhibitors of the mutant proteins. In particular, the Phase 3 ClarIDHy trial demonstrated a significant improvement in progression‐free survival (PFS, primary endpoint of the study) for patients with *IDH1* mutant CCA treated with the *IDH1* inhibitor ivosidenib in second or third line, as compared to placebo [2.7 months (95% CI, 1.6–4.2) vs. 1.4 months (95% CI, 1.4–1.6); hazard ratio (HR) 0.37, 95% CI, 0.25–0.54; *p* < 0.0001] [17]. In terms of OS, the final analysis showed a mOS of 10.3 months (95% CI, 7.8–12.4 months) with ivosidenib vs. 7.5 months (95% CI, 4.8–11.1 months) with placebo (HR: 0.79, 95% CI, 0.56–1.12; *p* = 0.09). Additionally, when adjusted for crossover with the rank‐preserving structural failure time (RPSFT) method, as pre‐planned in the study design, mOS with placebo was 5.1 months (95% CI, 3.8–7.6 months; HR: 0.49, 95% CI, 0.34–0.70; *p* < 0.001) [18]. Based on these positive results, ivosidenib received FDA and EMA approval in this setting.

However, given the rarity of the disease, there is few data yet regarding ivosidenib use in daily clinical practice, even if there are small case series published that confirm its activity in patients with pre‐treated *IDH1* mutant CCA [19, 20]. In this work, we aimed to evaluate ivosidenib efficacy and safety in a large real‐life cohort of patients and to provide a comparison with the efficacy of second‐line chemotherapy.

## 2 Materials and Methods

### 2.1 Study Population, Procedures and Objectives

This was an observational, retrospective, multicenter study conducted in 11 institutions based in Italy, Spain and Germany. Patients treated with ivosidenib for locally advanced or metastatic CCA carrying the *IDH1* mutation from May 2021 to September 2024 were included in the study. Clinical, pathological and molecular data were prospectively collected at the single institutions, pooled in a common dataset, and retrospectively analysed. All patients were followed up until death, loss of contact or time of data lock (01 November 2024). Ivosidenib was administered at the standard dose of 500 mg once daily in continuous 28‐day cycles.

NGS of the tumours was performed through the Oncomine Comprehensive Assay Plus or the FoundationOne CDx panel [21] as per routine local clinical practice.

The primary study objective was to investigate PFS in patients with *IDH1* mutant advanced CCA receiving ivosidenib after progression to at least one previous systemic treatment. Secondary objectives were to assess OS, Overall Response Rate (ORR), Disease Control Rate (DCR), and safety.

Finally, as pre‐planned explorative objectives, we evaluated the impact of molecular alterations found at the NGS on survival outcomes; we compared the efficacy of ivosidenib to that of second‐line standard chemotherapy with FOLFOX or CAPOX in a historical cohort [22]. The study cohort included 125 consecutive patients with *IDH1* mutated CCA treated between January 2013 and March 2021 across six Italian centres and one Spanish centre. Patients were included if they had resectable, locally advanced, or metastatic disease, and all cases were confirmed histologically as CCA with an *IDH1* mutation identified by next‐generation sequencing. Among the 125 patients analysed, 41 patients received second‐line treatment with XELOX or FOLFOX after progression on first‐line therapy; this subgroup was evaluated as the control cohort for comparison with patients treated with ivosidenib.

The study was approved by the Ethics Committees at each participating institution and was conformed to the ethical guidelines of the 1975 Declaration of Helsinki.

### 2.2 Statistical Analysis

Standard descriptive statistics were used to summarise clinical and biological patients' characteristics. Categorical variables were compared with Fisher's exact test. Median follow‐up was calculated through the reverse Kaplan –Meier method. The primary endpoint, PFS, was defined as the time from the beginning of treatment with Ivosidenib to disease progression or death. OS was defined as the time from the beginning of treatment with Ivosidenib to death from any cause. OS and PFS were estimated by the Kaplan–Meier method, and curves were compared by the log rank test. Patients who did not progress or die by the data cutoff date were censored at the last adequate assessment date. Treatment response was evaluated by computed tomography and categorised as complete response (CR), partial response (PR), stable disease (SD) or progressive disease (PD) by local review according to Response Evaluation Criteria in Solid Tumors (RECIST) 1.1. Imaging assessments were performed locally at each participating site and were not centrally reviewed.

ORR was defined as the rate of CR and PR under treatment with ivosidenib; DCR was defined as the rate of ORR plus the rate of SD under treatment with ivosidenib.

In addition, we evaluated the survival outcomes according to the genomic and molecular profile revealed by the NGS analysis.

We performed a statistical analysis to evaluate the baseline and genetic characteristics of patients treated with ivosidenib. Initially, a univariate analysis was conducted to identify potential associations between baseline clinical and genetic parameters and patient outcomes, including PFS and OS. Variables with a *p*‐value < 0.2 in the univariate analysis were subsequently included in a multivariate Cox proportional hazards regression model to adjust for potential confounders and identify independent prognostic factors. This approach allowed us to systematically assess the impact of each variable while accounting for their interdependence.

Finally, a safety analysis was conducted on all patients who received at least one dose of treatment, and adverse events (AEs) were graded using the National Cancer Institute Common Terminology Criteria for Adverse Events (NCI‐CTCAE) version 5.0 (9). A *p* value < 0.05 was considered statistically significant.

As a pre‐planned strategy to evaluate the impact of ivosidenib in the treatment strategy of CCA, we compared the second‐line outcomes of the study cohort with those of a previously published historical cohort of patients affected by advanced *IDH1* mutant CCA treated with second‐line FOLFOX/CAPOX [22].

For this aim, propensity score (PS) was calculated. All clinical and tumour variables available when treatment started were used for PS calculation to avoid incurring the possible imbalance of other parameters not correlated with the probability of receiving ivosidenib but with unknown effect on the outcome. The obtained PS was then used to generate stabilised inverse probability of treatment weights (IPTW) through appropriate math, which were used to weight each clinical feature, as well as measured outcomes, of each patient in both groups. After weighting baseline characteristics, *p*‐values were recalculated, and adequate balance was declared if all variables returned *p* > 0.05.

Once the weighted pseudo‐population of patients was obtained, differences between outcomes of ivosidenib and FOLFOX/CAPOX were analysed. IPTW‐adjusted Kaplan–Meier curves were calculated to graphically compare survivals among groups.

MedCalc package (MedCalc version 16.8.4) was used for statistical analysis.

## 3 Results

### 3.1 Patient Characteristics

Overall, 46 patients who received at least one dose of ivosidenib were included in the study.

The higher proportion of patients were female (63%) with a median age of 56 years (range 32–76), and 45.6% were submitted to primary tumour resection. All the patients received chemotherapy as first‐line treatment: 71.7% received cisplatin plus gemcitabine (CisGem), 17.4% received cisplatin plus gemcitabine plus durvalumab (CisGem Durva), whereas 10.9% received other chemotherapy regimens, including gemcitabine in monotherapy, gemcitabine plus oxaliplatin plus nab‐paclitaxel, and the doublet 5‐fluorouracil and irinotecan. Overall, 43.5% received ivosidenib as second‐line treatment, 34.8% as third‐line treatment, 15.2% as fourth‐line treatment and 6.5% beyond the fourth line. Nevertheless, it is worth noting that 48.8% of patients presented an excellent performance status assessed as Eastern Cooperative Oncology Group (ECOG) score of 0 before starting ivosidenib.

The complete baseline clinic‐pathological characteristics are reported in Table 1.

**TABLE 1:** Baseline clinicopathological characteristics of the study cohort, including demographics, prior treatments, performance status and disease characteristics.

Age	
≤ 70	33 (71.7%)
> 70	13 (28.3%)
Gender	
Male	17 (37.0%)
Female	29 (63.0%)
Histotype	
Small duct	46 (100%)
Large duct	0 (0%)
Localisation	
Intrahepatic	45 (97.8%)
Extrahepatic	1 (2.2%)
Previous surgery	
Yes	21 (45.6%)
No	25 (54.4%)
First‐line treatment	
CisGem	33 (71.7%)
CisGem Durva	8 (17.4%)
Other	5 (10.9%)
Pfs first‐line	
< 7 months	24 (52.2%)
≥ 7 months	22 (47.8%)
Response first‐line	
ORR	31 (67.4%)
PD	15/32.6%)
Line ivosidenib	
2	20 (43.5%)
3	16 (34.8%)
4	7 (15.2%)
> 4	3 (6.5%)
ECOG	
0	22 (48.8%)
> 0	24 (51.2%)
Markers (Ca19.9)	
NV	15 (51.7%)
> NV	14 (48.3%)
NLR	
< 3	14 (37.8%)
≥ 3	23 (62.2%)
Albumin	
NV	26 (81.2%)
No NV	6 (18.8%)
AST	
NV	19 (48.7%)
No NV	20 (51.3%)
ALT	
NV	29 (74.3%)
No NV	10 (25.7%)

### 3.2 Survival Outcomes

The median follow‐up was 11.9 months (95% CI, 1.09–23.2). At the data cutoff, 25 patients (54.3%) had died, and 39 patients (84.8%) discontinued the treatment due to disease progression. In our cohort, the median PFS during first‐line treatment was 7.0 months (95% CI, 4.3–11.8), and this value was therefore used as the cutoff point for PFS‐based analyses.

**FIGURE 1:**
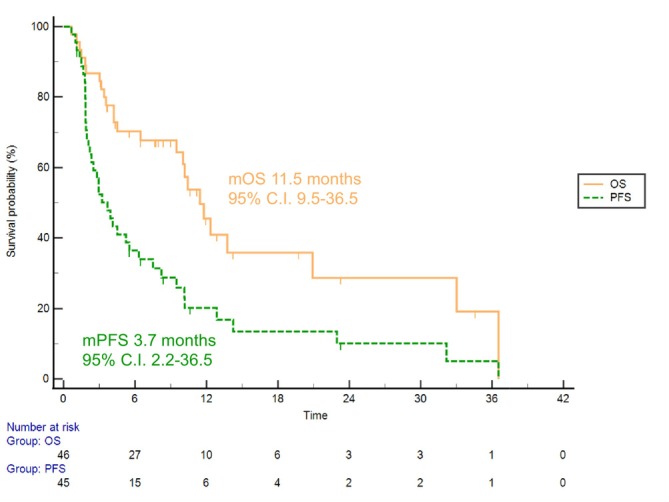
Progression‐free survival (PFS) and overall survival (OS) from the start of ivosidenib treatment, regardless of treatment line.

**FIGURE 2:**
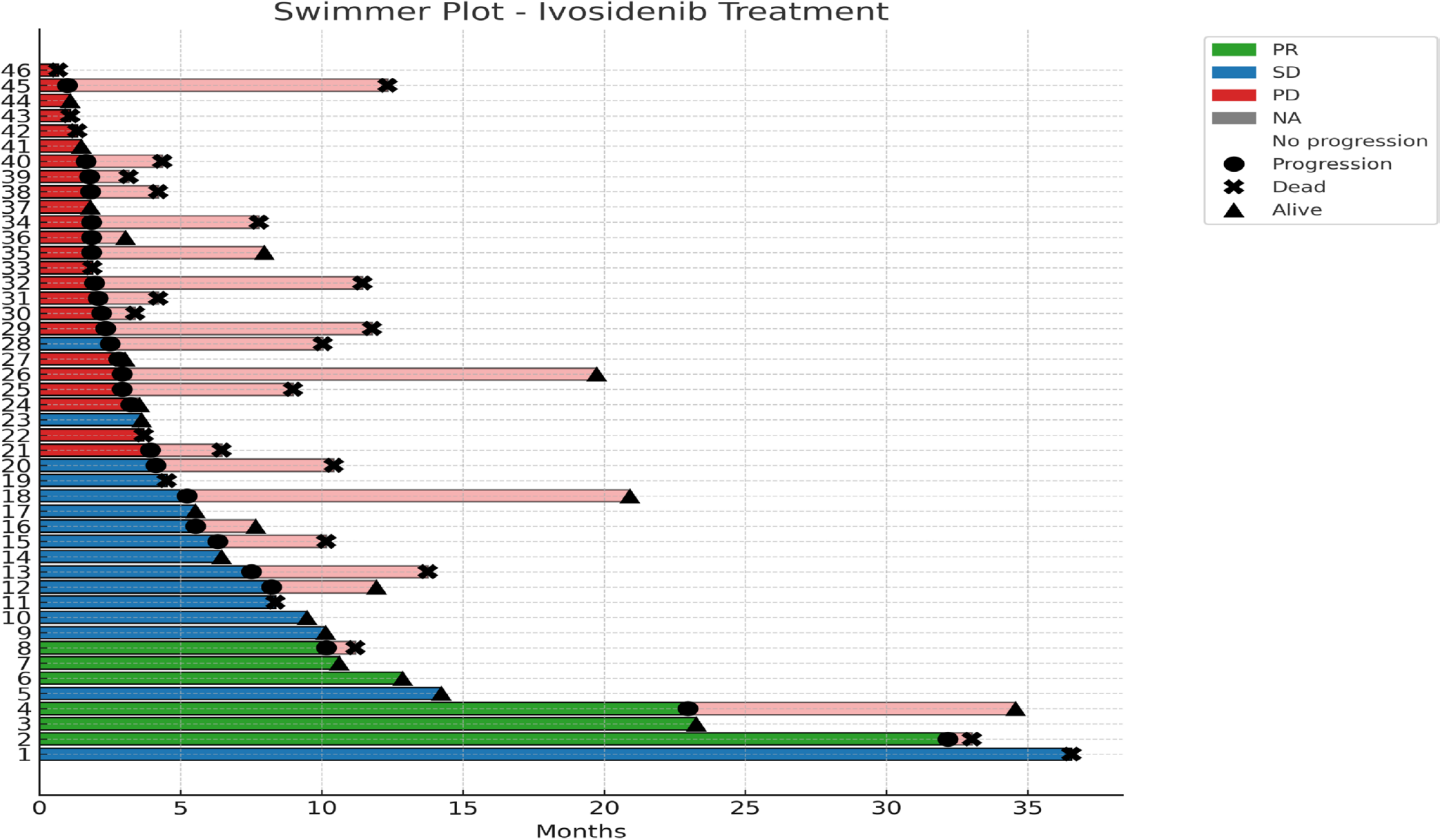
Swimmer plot of progression‐free survival (PFS) and post‐progression survival in patients treated with ivosidenib. Each bar represents an individual patient. Green, blue, red and grey bars indicate best overall response as partial response (PR), stable disease (SD), progressive disease (PD) and not available (NA), respectively. Light red segments represent survival time following progression. Vertical bars (|) indicate patients with no progression (censored at last follow‐up), while circles (o) indicate patients who experienced progression. The symbol X denotes patients who were deceased at last follow‐up, and triangles (▲) indicate patients alive at last follow‐up.

Overall, mPFS was 3.7 months (95% CI, 2.2–36.5), while mOS from the start of ivosidenib was 11.5 months (95% CI, 9.5–36.5) across all treatment lines (Figure 1). In terms of investigator‐assessed response, data were available for 44 patients; DCR was 50.0%, with 13.6% of patients achieving a PR. The swimmer plot illustrates the duration of treatment and the post‐progression period for each patient treated with ivosidenib (Figure 2).

**FIGURE 3:**
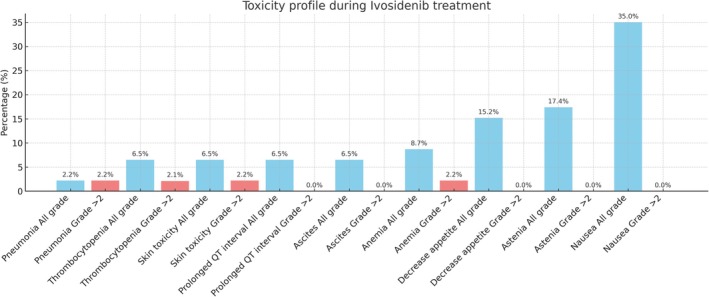
Safety data assessment in the overall patient population.

Safety data were available for all patients (Figure 3). Any grade AEs occurred in 69.6% of patients. Grade 3 and 4 AEs occurred in 8.7% of patients. The most common AEs were nausea (35.0%), asthenia (17.4%) and decreased appetite (15.2%). No treatment‐related deaths were reported.

**FIGURE 4:**
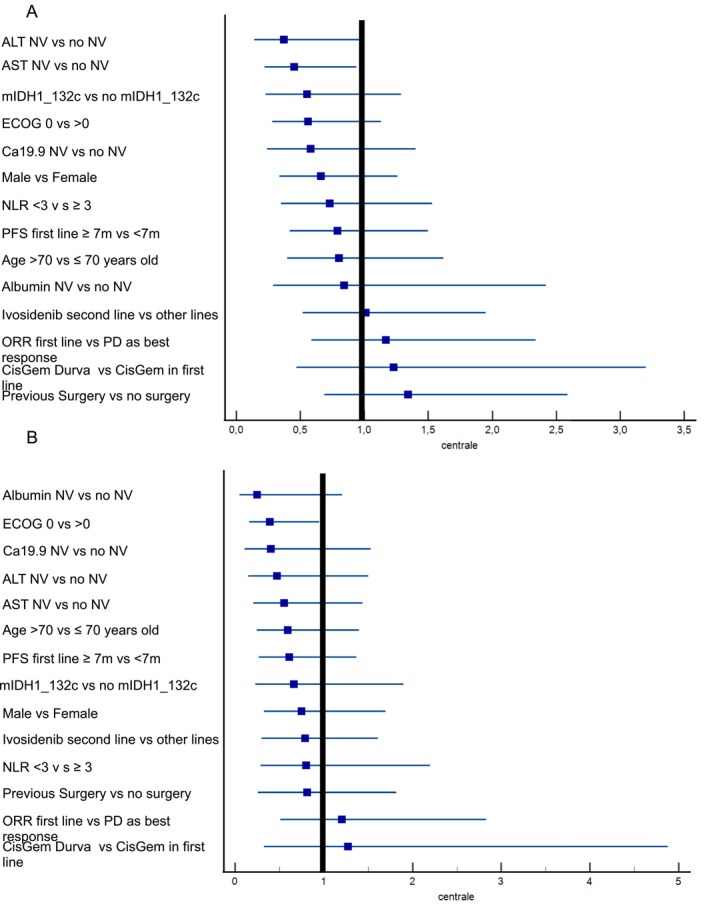
Forest plot depicting the univariate analysis of progression‐free survival (A) and overall survival (B) according to baseline characteristics.

As highlighted in the forest plot depicted in Figure 4A, in univariate analysis, ivosidenib resulted in a better PFS in patients with normal aspartate transaminase and with normal alanine aminotransferase. No other baseline characteristic, including the type of *IDH1* mutation, the line of ivosidenib administration, the response or duration of response to the first‐line, and the type of first‐line treatment, was found to be correlated with a different prognosis. In terms of OS, ivosidenib showed a better outcome only in patients with ECOG 0 compared to patients with ECOG > 0 (Figure 4B). These findings were further confirmed in the multivariate analysis, where no additional significant prognostic factors were identified beyond those observed in the univariate analysis.

Noteworthy, mOS was correlated with the best treatment response. Specifically, patients with PR had a mOS of 33.1 months (95% CI, 33.1–33.1); those with SD had a mOS of 13.7 months (95% CI, 9.8–36.5), and patients with PD had a mOS of 4.2 months (95% CI, 3.0–11.4) (Figure S1).

Furthermore, in this cohort study, 15.2% (*N* = 7) of patients who received ivosidenib remained on treatment for one year or longer. Of them, 71.4% (*N* = 5) had PR and 28.6% (*N* = 2) had a SD as best response.

From the molecular point of view, data from NGS analysis were available for 15/46 patients (32.6%). As depicted in Figure S2, the most frequently altered genes in this population were *BAP1* (40%, *N* = 6), *TP53* and *CDKN2A/B* (26%, *N* = 4). The forest plot for OS did not highlight any gene associated with survival in patients treated with ivosidenib, while for PFS, patients with alterations in *CDKN2A/B* and *MTAP* showed a worse prognosis (Figure S3).

### 3.3 Efficacy of Ivosidenib Compared to FOLFOX/CAPOX in Second Line

As a pre‐planned exploratory analysis to evaluate the impact of ivosidenib in the treatment strategy of CCA, we compared the second‐line outcomes of the study cohort with those of a previously published historical cohort of patients affected by advanced *IDH1* mutant CCA treated with second‐line FOLFOX/CAPOX.

Overall, 61 patients were enrolled: 20 patients treated with ivosidenib and 41 patients treated with FOLFOX/CAPOX. No differences were found between the two study cohorts in terms of baseline characteristics (Table 2).

**TABLE 2:** Comparison of baseline characteristics between patients treated with ivosidenib and those treated with FOLFOX/CAPOX in the second‐line setting, before and after IPTW‐adjustment.

	Before IPTW‐adjustment			After IPTW‐adjustment		
	IVOSIDENIB (*n* = 20)	FOLFOX/XELOX (*n* = 41)	*p*	IVOSIDENIB (*n* = 20)	FOLFOX/XELOX (*n* = 41)	*p*
Age						
≤ 70	15 (75.0%)	29 (70.7%)	0.77	16 (70.0%)	28 (68.3%)	0.38
> 70	5 (25.0%)	12 (29.3%)		4 (30.0)	13 (31.7%)	
Gender						
Male	6 (30.0%)	14 (34.1%)	1.00	6 (30.0%)	15 (36.6%)	0.77
Female	14 (70.0%)	27 (65.9%)		14 (70.0%)	26 (63.4%)	
Previous surgery						
Yes	9 (45.0%)	14 (34.1%)	0.57	9 (45.0%)	18 (43.9%)	0.58
No	11 (55.0%)	27 (65.9%)		11 (55.0%)	23 (56.1%)	
Pfs first‐line						
< 7 months	11 (55.0%)	20 (48.8%)	0.78	10 (50.0%)	22 (53.6%)	1.00
≥ 7 months	9 (45.0%)	21 (51.2%)		10 (50.0%)	19 (46.4%)	
Response to first‐line						
ORR	15 (75.0%)	28 (68.3%)	0.76	16 (70.0%)	29 (70.7%)	0.54
PD	5 (25.0%)	13 (31.7%)		4 (30.0%)	12 (29.3%)	
ECOG						
0	11 (55.0%)	20 (48.8%)	0.78	9 (45.0%)	21 (51.2%)	1.00
> 0	9 (45.0%)	21 (51.2%)		11 (55.0%)	20 (48.8%)	
Marker (Ca19.9)						
NV	12 (60.0%)	23 (56.1%)	0.29	11 (55.0%)	20 (48.8%)	0.78
> NV	8 (40.0%)	18 (43.9%)		9 (45.0%)	21 (51.2%)	
NLR						
< 3	7 (35.0%)	14 (34.1%)	1.00	8 (40.0%)	14 (34.1%)	0.77
> 3	13 (65.0%)	27 (65.9%)		12 (60.0%)	27 (45.9%)	
Albumin						
NV	16 (80.0%)	29 (70.7%)	0.54	14 (70.0%)	28 (68.3%)	1.00
No NV	4 (20.0%)	12 (29.3%)		6 (30.0%)	13 (31.7%)	
AST						
NV	9 (45.0%)	22 (53.7%)	0.59	10 (50.0%)	21 (51.2%)	1.00
No NV	11 (55.0%)	19 (46.3%)		10 (50.0%)	20 (48.8%)	
ALT						
NV	15 (75.0%)	26 (63.4%)	0.40	15 (75.0%)	28 (68.3%)	0.76
No NV	5 (25.0%)	15 (36.6%)		5 (25.0%)	13 (31.7%)	

**FIGURE 5:**
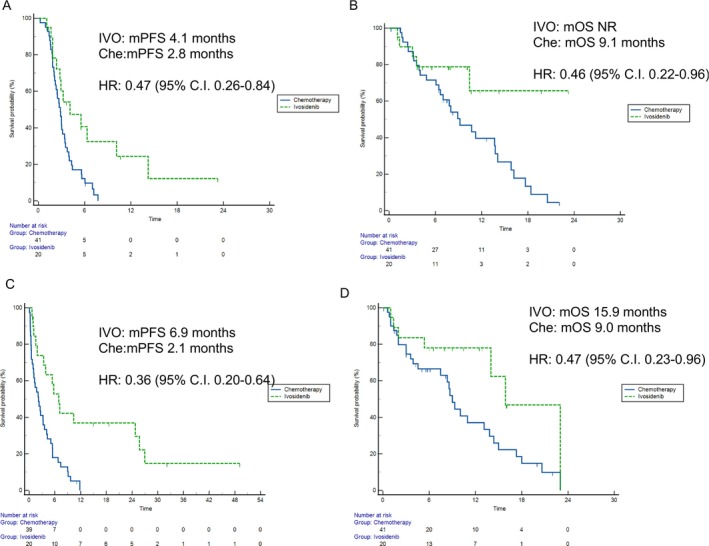
Comparison of second‐line outcomes between patients treated with ivosidenib and those treated with FOLFOX/CAPOX in a historical cohort. (A) Univariate analysis of progression‐free survival (PFS). (B) Univariate analysis of overall survival (OS). (C) Inverse probability of treatment weighting (IPTW)‐adjusted analysis of PFS. (D) IPTW‐adjusted analysis of OS.

At the univariate analysis for PFS, ivosidenib was found to have an impact, with mPFS of 4.1 months versus 2.8 months (HR: 0.47, 95% CI, 0.26–0.84, *p* = 0.01) in patients who received ivosidenib and FOLFOX/CAPOX, respectively (Figure 5A).

At the univariate analysis for OS, ivosidenib was found to have a prognostic impact, with mOS not reached versus 9.1 months (HR: 0.46, 95% CI, 0.22–0.96, *p* = 0.04) in patients who received ivosidenib and FOLFOX/CAPOX, respectively (Figure 5B).

Ivosidenib showed a tendency toward higher PR, SD and DCR, which did not reach statistical significance (*p* = 0.25, *p* = 0.27 *p* = 0.15, respectively) (Figure S4).

After IPTW‐adjustment, baseline clinical and tumour characteristics were similar between the two groups (Table 2), as indicated by a *p*‐value > 0.05 in all cases.

In this population, mPFS (Figure 5C) was 6.9 months with ivosidenib and 2.1 months with FOLFOX/CAPOX (HR: 0.36, 95% CI, 0.20–0.64, *p* = 0.0005); mOS (Figure 5D) with ivosidenib was 15.9 and 9.0 months with FOLFOX/CAPOX (HR: 0.47, 95% CI, 0.23–0.96, *p* = 0.0405).

## 4 Discussion

Real‐life studies are particularly relevant since the profile of patients in daily clinical practice may differ from what is observed in randomised Phase 3 studies. There is limited real‐world data regarding the efficacy and safety of ivosidenib for patients affected by *IDH1* mutant advanced CCA, and it is crucial to bridge this gap mainly for two reasons: (a) the ClarIDHy trial showed a relatively small, albeit statistically significant, absolute gain in terms of PFS compared to placebo; (b) an appropriate comparator was not available when the study was designed [2]. However, following the results of the ABC‐06 trial, FOLFOX has now emerged as the standard second‐line treatment.

To the best of our knowledge, our study is the first, outside of clinical trials and small case series, to confirm the efficacy and safety of ivosidenib as second and further line treatment in a large international cohort of patients with advanced *IDH1* mutant CCA and to provide a comparison to the efficacy of second‐line chemotherapy, designed to fill the previously mentioned gaps.

In terms of patients' characteristics, it is worth noting that (a) all patients in our study had metastatic disease, which tends to have least options and worst prognosis compared to locally advanced disease [23, 24]; (b) almost 60% of our patients received ivosidenib as third or further line (while in the Phase 3 trial 52% of patients were treated in second line and no patient was treated beyond third line as per study design). Despite this, ivosidenib proved to be active in our cohort, with a mPFS of 3.7 months, an ORR of 13.6% and, most notably, with a mOS of 11.5 months. Even if the response rate gained with ivosidenib is lower than what we see with other targeted therapies for advanced CCA (e.g., FGFR inhibitors [25, 26], with up to 42% ORR), our study confirms that the clinical benefit from *IDH* inhibition can be durable. Indeed, our cohort had a small but not negligible number of patients (*N* = 7, 15%) who remained on treatment with ivosidenib for one year or longer, and a strong correlation between best response and mOS was shown.

When we put these results in context, all outcomes in our cohort were in line not only with that of the ClarIDHy study, but also with historical data of second‐line treatments and of the ABC‐06 study [2, 7, 19], even if most of our patients were treated in third or further line.

Furthermore, since the ClarIDHy study was a placebo‐controlled study and no direct comparison between ivosidenib and second‐line chemotherapy is available, we conducted a pre‐planned comparison between patients treated with ivosidenib as second‐line treatment in our cohort and a previously published cohort of patients affected by advanced *IDH1* mutant CCA treated with second‐line FOLFOX/CAPOX.

There is conflicting evidence regarding the prognostic role of *IDH1* mutations for CCA [27, 28, 29, 30] and of its predictive impact in terms of response to standard chemotherapy; preclinical evidence indicated that mutant *IDH1/2*‐induced increase of 2HG leads to changes in DNA repair pathways [31, 32, 33, 34] and to homologous recombination deficiency (HRD). This evidence suggested that *IDH* mutant CCA could benefit from DNA damaging agents, but this hypothesis was not confirmed by clinical studies [35, 36]. In particular, in terms of platinum‐based chemotherapy, our previous study showed that patients with *IDH1* mutant CCA did not have improved outcomes with second‐line FOLFOX/CAPOX compared to patients with *IDH1* wild type CCA [37]. To the best of our knowledge, the current study is the first to suggest that ivosidenib may achieve a more favourable outcome compared to chemotherapy in this setting, thus suggesting that the use of this targeted agent in the second‐line setting may be the preferable strategy.

Additionally, it has to be taken into account that the treatment landscape of CCA has changed with the introduction of PD‐(L)1 inhibitors in combination with cisplatin and gemcitabine [4, 5] and that most data about the prognostic and predictive impact of *IDH1* mutations on second and further line treatments do not include patients treated with the new first‐line standard of care. This is particularly relevant since *IDH* mutations were shown to have an immunosuppressive effect, both in CCA and other tumours [38, 39], but so far this was not confirmed in subgroup analyses of clinical trials [40] nor in real‐world data [41], where *IDH1* mutations were not associated with a different outcome of chemo‐immunotherapy.

In this context, even if our cohort mostly included patients treated with CisGem as first‐line therapy, a small subset of patients were treated with chemo‐immunotherapy before ivosidenib. Even with the limitations of a small cohort, there were no significant differences in the outcome on ivosidenib in this group.

Finally, while no specific co‐occurring gene alteration was associated with a significant impact on OS in patients treated with ivosidenib, *CDKN2A/B* and *MTAP* alterations correlated with a worse PFS, suggesting a potential role in resistance to *IDH1* inhibition or even the potential to combine *IDH1* inhibitors with MTA‐cooperative PRMT5 inhibitors in *MTAP* deleted tumours to synergistically target different metabolic vulnerabilities.

This study has some clear limitations: first, it is a retrospective, observational study with a limited sample size; the absence of a randomised control group prevents direct comparisons between ivosidenib and other second‐line treatments. Furthermore, given the retrospective nature of this study, the accuracy of response categorisation between PR and SD may be subject to potential bias; as imaging assessments were performed locally and were not centrally reviewed, inter‐observer variability may have further influenced the accuracy of response evaluation. However, we chose to maintain this distinction in the survival analyses to allow for a more direct comparison with the phase III registrational trial, which reported outcomes separately for these subgroups. Additionally, the relatively limited sample size of our cohort may have reduced the statistical power of certain analyses, particularly in subgroup comparisons, potentially limiting the ability to detect significant associations across all parameters explored. Finally, molecular data were available only for a small subset of patients, preventing definitive conclusions regarding the prognostic and predictive role of co‐occurring alterations.

In conclusion, despite its limitations, this study reinforces the role of ivosidenib as a viable treatment option for patients with metastatic *IDH1* mutant CCA, demonstrating consistent efficacy and a manageable safety profile. To further improve outcomes by refining patients' selection and developing new therapeutic strategies, future research should focus on unveiling the mechanisms driving primary and acquired resistance.

## Author Contributions

M.N., A.C.‐G.: conceptualisation. M.R., F.C., A.M., M.D.R., T.P., D.L., C.P., G.A., T.A., T.S., N.S., P.C., A.P., E.T.‐B., L.A., K.S.V., T.J.E., L.P., T.M.: Provision of study materials or patients. M.R., F.C., A.M., M.D.R., T.P., D.L., C.P., G.A., T.A., T.S., N.S., P.C., A.P., E.T.‐B., L.A., K.S.V., T.J.E., L.P., T.M.: collection and assembly of data. A.C.‐G.: formal analysis. M.N., A.C.‐G., C.S., S.C., M.P., S.F.: writing – original draft preparation. M.N., A.C.‐G., F.P., L.R., S.L., V.M.: writing – review and editing. All authors: final approval of manuscript.

## Ethics Statement

This study was conducted in accordance with the Declaration of Helsinki and received ethical approval from the Ethics Committee of each Institution. Informed consent was obtained from all participants prior to their involvement. Participant data will be kept confidential and stored securely. There are minimal risks associated with participation, and participants will benefit from the knowledge gained from this research.

## Conflicts of Interest

M.N. reported receiving travel expenses from AstraZeneca, speaker honoraria from Accademia della Medicina, Incyte and Servier; honoraria from Sandoz, Medpoint SRL, Incyte, AstraZeneca and Servier for editorial collaboration. Consultant honoraria from EMD Serono, Basilea Pharmaceutica, Incyte, MSD Italia, Servier, AstraZeneca and Taiho. L.R. reported receiving consulting fees from AbbVie, AstraZeneca, Basilea, Bayer, BMS, Eisai, Elevar Therapeutics, Exelixis, Genenta, Hengrui, Incyte, Ipsen, IQVIA, Jazz Pharmaceuticals, MSD, Nerviano Medical Sciences, Roche, Servier, Taiho Oncology, Zymeworks; lecture fees from AstraZeneca, Bayer, BMS, Guerbet, Incyte, Ipsen, Roche, Servier; travel expenses from AstraZeneca and Servier; institutional research funding from AbbVie, Agios, AstraZeneca, BeiGene, Eisai, Exelixis, Fibrogen, Incyte, Ipsen, Jazz Pharmaceuticals, Lilly, MSD, Nerviano Medical Sciences, Roche, Servier, Taiho Oncology, TransThera Sciences, Zymeworks. S.L. reported receiving personal honoraria as an invited speaker from Amgen, Astra Zeneca, Bristol‐Myers Squibb, Incyte, GSK, Lilly, Merck Serono, MSD, Pierre‐Fabre, Roche and Servier; participation in an advisory board for Amgen, Astellas, Astra Zeneca, Bayer, Bristol‐Myers Squibb, Daiichi‐Sankyo, GSK, Incyte, Lilly, Merck Serono, MSD, Servier, Takeda, Rottapharm, Beigene, Fosun Pharma and Nimbus Therapeutics. F.P. reported receiving research funding (to Institution) from Lilly, BMS, Incyte, AstraZeneca, Amgen, Agenus, Rottapharm; personal honoraria as an invited speaker from BeiGene, Daiichi‐Sankyo, Seagen, Astellas, Ipsen, AstraZeneca, Servier, Bayer, Takeda, Johnson & Johnson, BMS, MSD, Amgen, Merck‐Serono, Pierre‐Fabre. Advisory/consultancy from BMS, MSD, Amgen, Pierre‐Fabre, Johnson & Johnson, Servier, Bayer, Takeda, Astellas, GSK, Daiichi‐Sankyo, Pfizer, BeiGene, Jazz Pharmaceuticals, Incyte, Rottapharm, Merck‐Serono, Italfarmaco, Gilead, AstraZeneca, Agenus. L.P. reported receiving Advisory/Consultancy from Servier, Astellas, Jazz Pharmaceuticals, AstraZeneca. THE reported receiving Advisory/Consultancy from: MSD, Roche, Sanofi, BMS, AstraZeneca, MSD, Pierre Fabre, Servier, Lilly, Ipsen, Daiichi Sankyo, Takeda. The other authors declare no conflicts of interest.

## Supporting information


**Figure S1:** Genomic landscape of patients treated with ivosidenib, showing the most frequently altered genes identified through next‐generation sequencing (NGS).

**Figure S2:** Correlation between best treatment response and median overall survival (mOS), illustrating differences in survival outcomes based on response categories.

**Figure S3:** Forest plot of the impact of genomic alterations on survival outcomes, highlighting potential associations between specific gene alterations and progression‐free survival (PFS) or overall survival (OS).

**Figure S4:** Best response rates in patients receiving ivosidenib versus FOLFOX/CAPOX in second line, comparing partial response and stable disease rates between the two groups.

## Data Availability

The data that support the findings of this study are available on request from the corresponding author. The data are not publicly available due to privacy or ethical restrictions.
