# Supplementary figures and images for: Ivosidenib for IDH1‐Mutant Intrahepatic Cholangiocarcinoma: Insights From a Multicenter Real‐World Study
Source: Liver Int. 2025 Aug 26;45(9):e70295. doi: 10.1111/liv.70295 (PMC12379572; doi:10.1111/liv.70295)

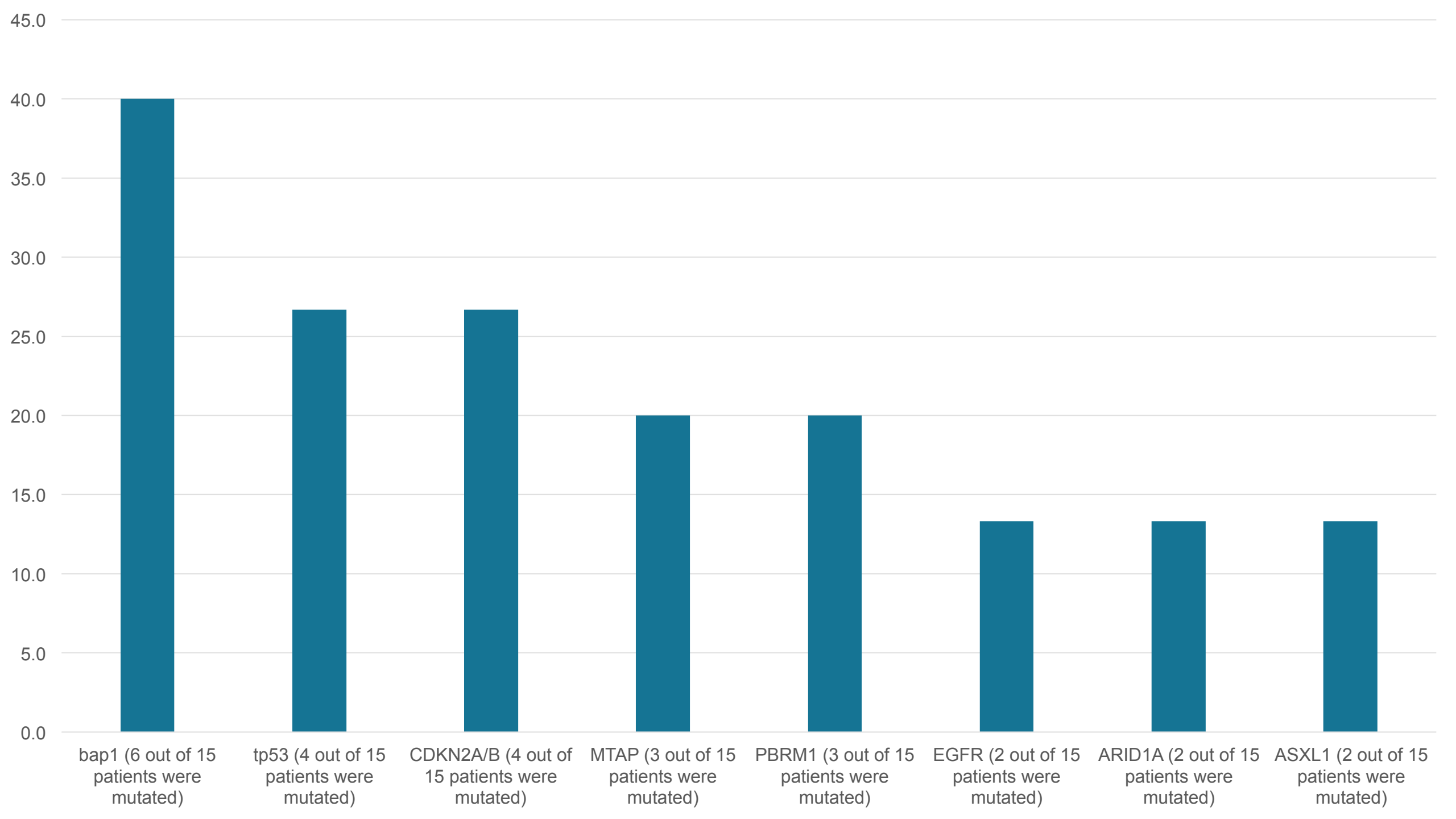

Supplement: Supplementary file 1 — Figure S1: Genomic landscape of patients treated with ivosidenib, showing the most frequently altered genes identified through next‐generation sequencing (NGS). [file LIV-45-0-s003.pdf]

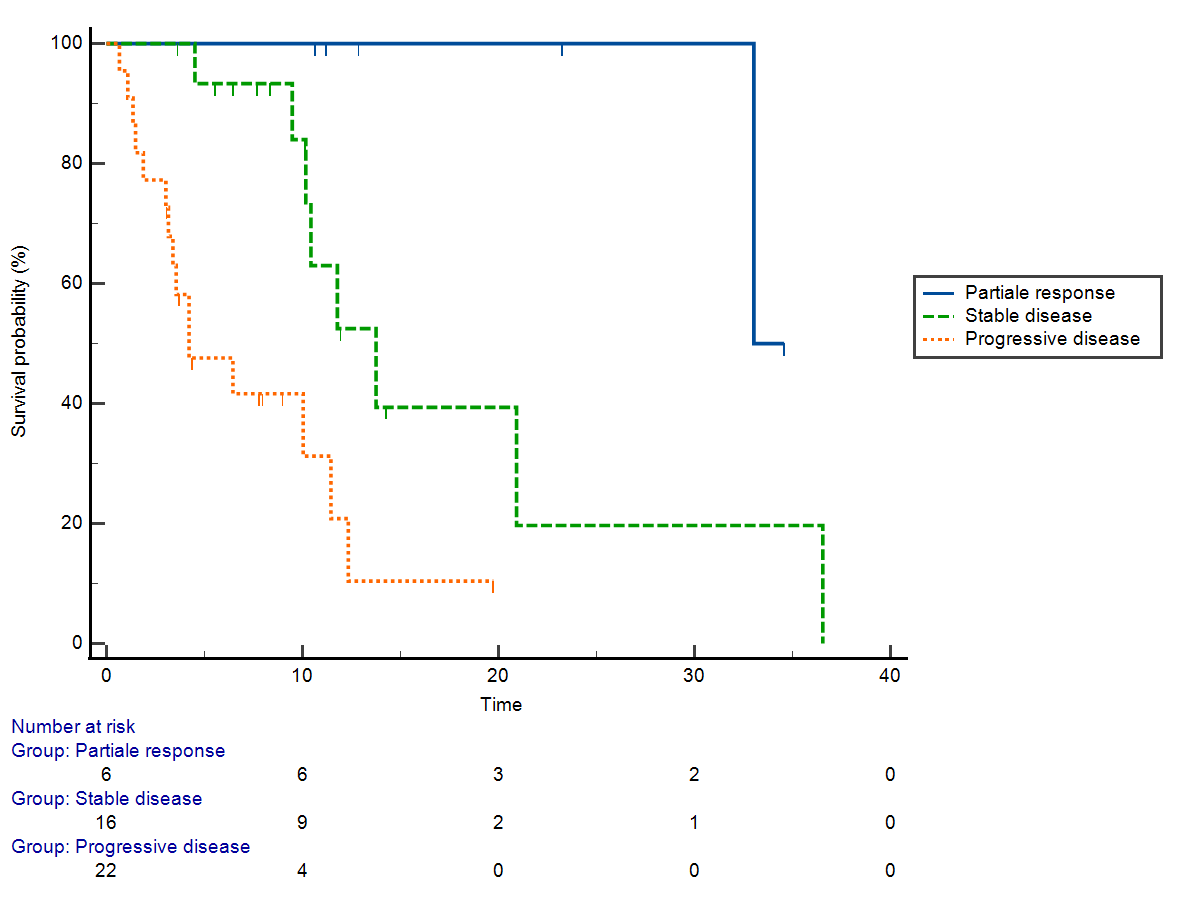

Supplement: Supplementary file 2 — Figure S2: Correlation between best treatment response and median overall survival (mOS), illustrating differences in survival outcomes based on response categories. [file LIV-45-0-s001.png]

A

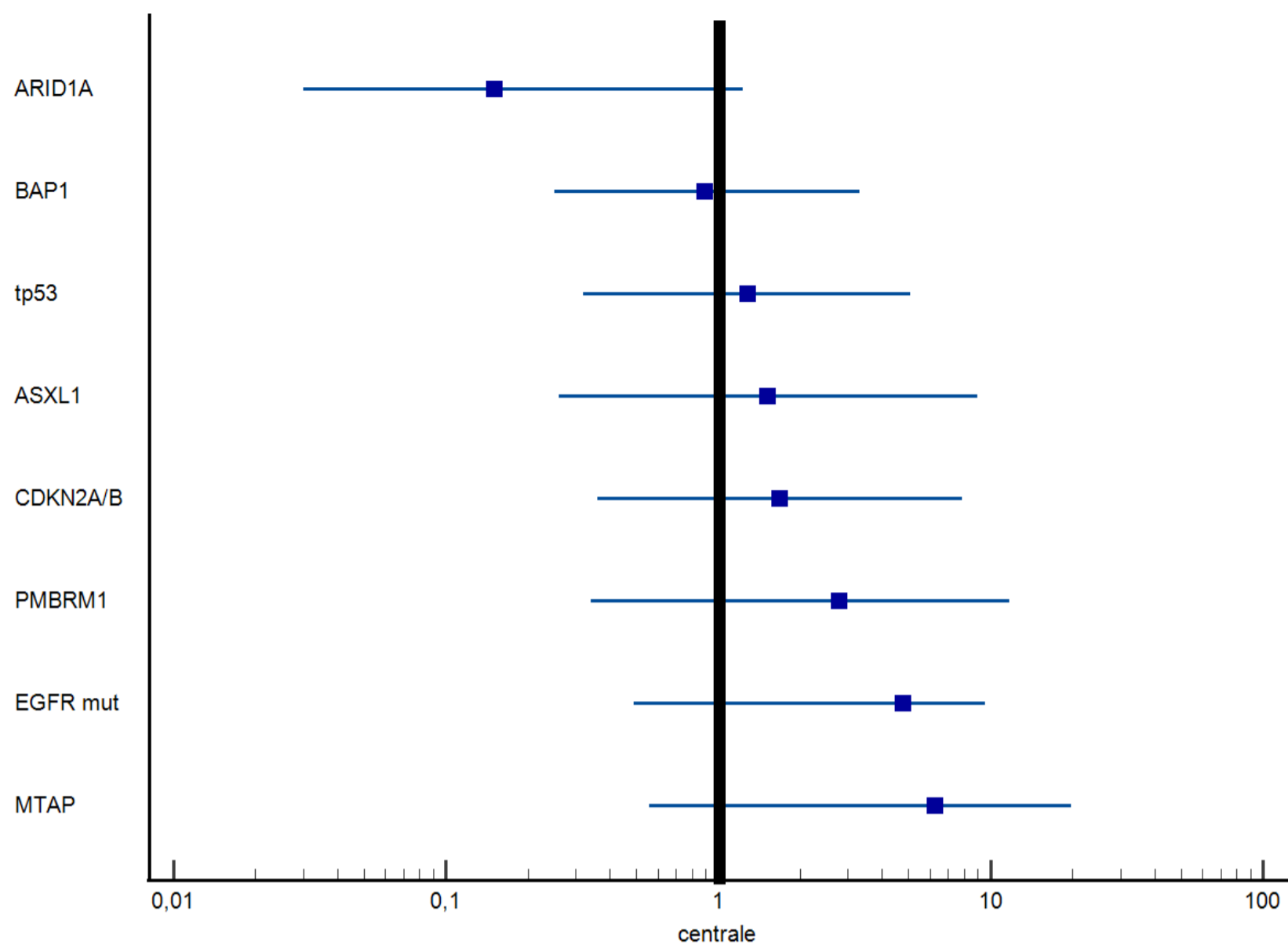

B

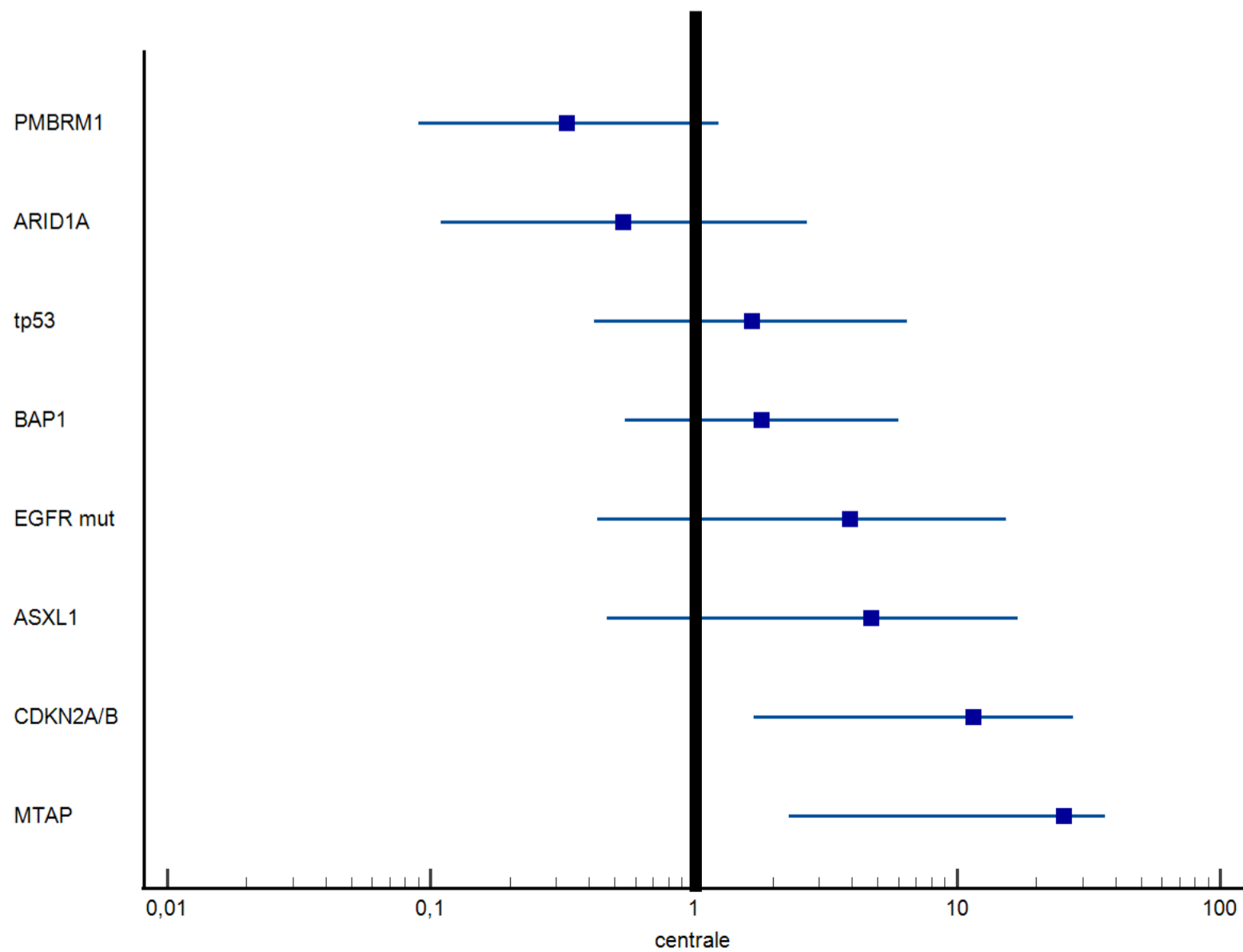

Supplement: Supplementary file 3 — Figure S3: Forest plot of the impact of genomic alterations on survival outcomes, highlighting potential associations between specific gene alterations and progression‐free survival (PFS) or overall survival (OS). [file LIV-45-0-s004.pdf]

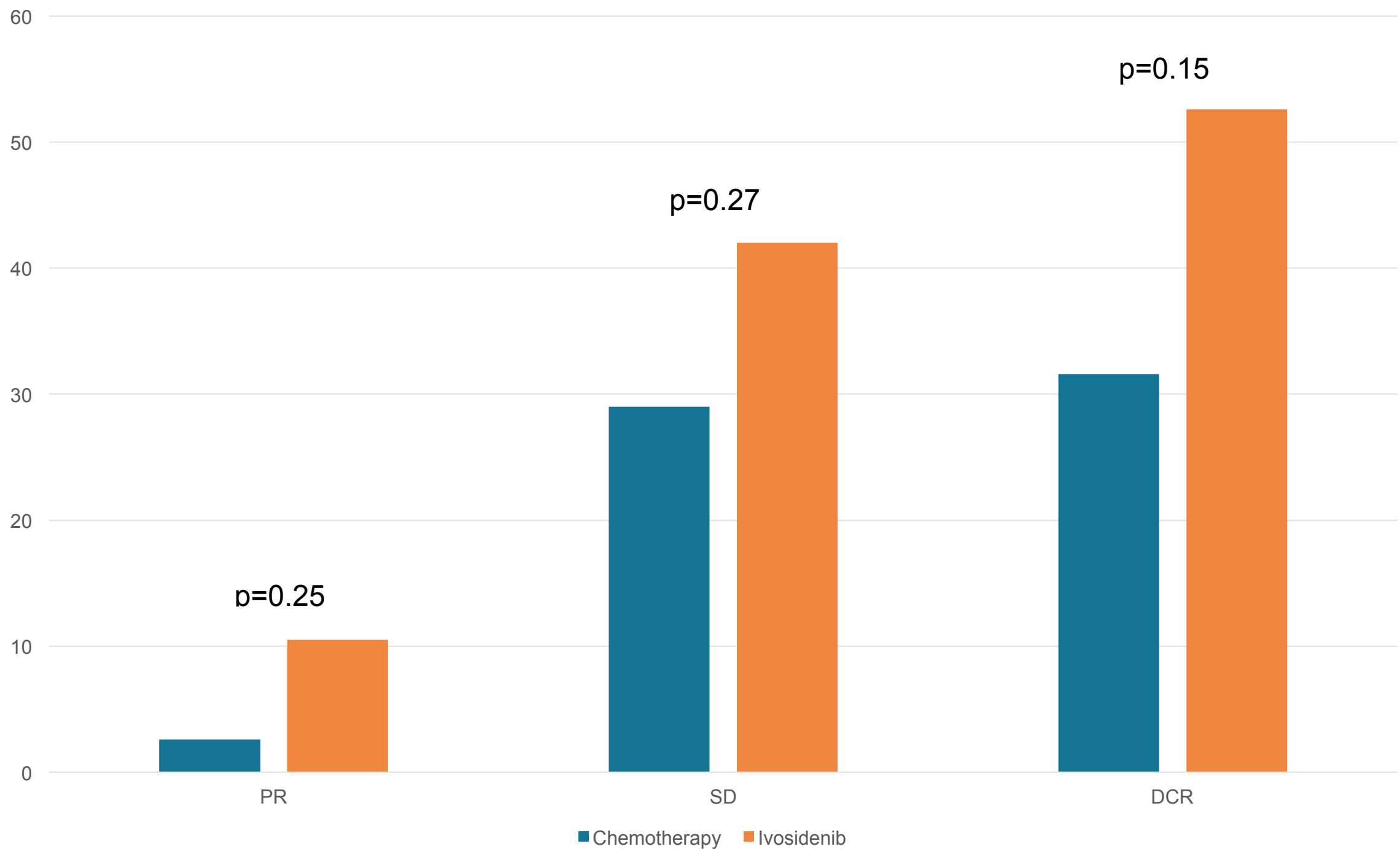

Supplement: Supplementary file 4 — Figure S4: Best response rates in patients receiving ivosidenib versus FOLFOX/CAPOX in second line, comparing partial response and stable disease rates between the two groups. [file LIV-45-0-s002.pdf]
